# Computational Transcendence: Responsibility and agency

**DOI:** 10.3389/frobt.2022.977303

**Published:** 2022-09-26

**Authors:** Jayati Deshmukh, Srinath Srinivasa

**Affiliations:** International Institute of Information Technology, Bangalore, India

**Keywords:** responsible ai, multi-agent systems, AI ethics, autonomy, agency, identity

## Abstract

Emergence of responsible behavior is explored in non-cooperative games involving autonomous agents. Rather than imposing constraints or external reinforcements, agents are endowed with an elastic “sense of self” or an *elastic identity* that they curate based on rational considerations. This approach is called “computational transcendence (CT).” We show that agents using this model make choices for collective welfare instead of individual benefit. First, relevance of this model in game theoretic contexts like Prisoners’ dilemma and collusion is presented. Next, a generic multi-agent framework for simulating dilemmas around responsible agency is also proposed. CT implemented on this framework, is shown to be versatile in acting responsibly to different kinds of circumstances–including modifying their strategy based on their interaction with other agents in the system as well as interacting with adversaries that are rational maximizers, and who have a rationale to exploit responsible behavior from other agents. CT is also shown to outperform reciprocity as a strategy for responsible autonomy. Thus, we present CT as a framework for building autonomous agents which can intrinsically act responsibly in multi-agent systems. The core model for computational ethics presented in this paper can potentially be adapted to the needs of applications in areas like supply chains, traffic management, and autonomous vehicles. This paper hopes to motivate further research on responsible AI, by exploring computational modeling of this elusive concept called the “sense of self” that is a central element of existential inquiry in humans.

## 1 Introduction

Responsible behavior is often in conflict with self-interest driven autonomous agency Rapoport et al. (1965); Axelrod and Hamilton (1981). This is true of both humans and autonomous artificial intelligence (AI). Reconciling between the pursuit of individual self-interest, and one’s social responsibility have elicited a lot of philosophical inquiry since millennia. As autonomous AI makes fast inroads into our collective lives, the need for designing *computational foundations* for responsible behavior is becoming more urgent Matthias (2004). The problem is exacerbated in multi-agent systems where several autonomous agents interact in a shared environment, resulting in complex emergent conditions, whose implications may not be *a priori* apparent.

In this paper, we define *responsible* behaviour by an autonomous agent as selecting an action which is aligned towards collective welfare even in the presence of lucrative alternate but *irresponsible* actions, which are individually beneficial (and hence, rational) for the agent. In this context, we propose a model which leads to emergent responsible behaviour in autonomous agents even where they are tempted with irresponsible choices.

Machine ethics is gaining prominence in recent times, and ethical dilemmas addressed from an individual agent’s perspective have resulted in several variants of the well-known “Trolley problem” Bonnefon et al. (2016); Bruers and Braeckman (2014); Edmonds (2013); Goodall (2014). The idea of ethics is largely a philosophical debate with multiple paradigms defining what is or ought to be termed ethical. However, there is an emerging consensus on the need for *responsible* AI Clarke (2019); Dignum (2017); Peters et al. (2020); Ramchurn et al. (2021); Yazdanpanah et al. (2021); Dastani and Yazdanpanah (2022); Dignum (2020); Allen et al. (2006); Gogoshin (2021), that is concerned with more tractable issues like upholding integrity, safety, explainability, accountability, and other systemic constructs within an overarching legal or ethical framework, rather than questioning the foundations of the framework itself.

However, even when there is clarity on what ought to be called responsible behavior, enforcing this is hard when agents act autonomously and interact with one another. Interacting autonomous agents trying to maximize self-interest, can lead to conditions like the prisoners’ dilemma Rapoport et al. (1965), where individual benefit and collective good are in conflict. Identifying such conditions at design time, and/or enforcing corrective actions in real-time, pose major challenges. There is hence a need to imbibe an *innate* sense of responsibility into models of agency.

Approaches to multi-agent ethics have also been addressed through various means like introducing morality as a separate explicit consideration that agents balance with their desires, and with heuristics representing responsible multi-agent interactions Cointe et al. (2016, 2020); Lorini (2012).

A common design feature in current approaches to agent morality is that, moral considerations are introduced as an extraneous construct in the agent model, that needs to be executed in addition to the logic of self-interest based utility maximization. In this work, we argue that moral considerations are not separate from self-interest and that moral reasoning can be a *direct consequence* of rational self-interest, with a slightly modified form of the core agent model.

Human societies are replete with examples where morality and responsible behavior *emerge* from affected populations, even in times of extreme conflict and oppression. There are many examples where human societies have been *more* cooperative rather than less, in times of crises, where formal structures of fair statehood and public morality have *emerged* as a bottom-up phenomenon Bregman (2020); Bandura (2006); Hamlin (2013).

Such emergence of public morality is often explained using extraneous constructs like reciprocity, empathy, altruism, virtue, and so on, that the population is thought to have imbibed. However, we argue that the emergence of public morality is a consequence of how individuals curate their “sense of self” or *identity*. Individuals identifying with other individuals or even abstract concepts, makes them internalize the interests of the object of their identity, into their own sense of utility. Curating the identity of agents, helps us distinguish “associations of identity” from “rational associations” that are conventionally studied. We show how an elastic sense of self can naturally lead to a sense of responsibility in self-interested agents. We also propose a model to show how the logic behind identity associations can itself be based on self-interest considerations, thus obviating the need for any independent concept like virtue or reciprocity, to be brought in to explain agent morality.

The key contributions of this paper are as follows: 1) We propose a bottom-up model called elastic sense of self for autonomous agents which we posit leads to responsible behaviour. 2) We demonstrate that an elastic sense of self in game-theoretic contexts like prisoners’ dilemma and collusion leads to collective good even when there is an incentive to act selfishly. 3) We propose a generic multi-agent framework for simulating dilemma between rational and responsible choices, and evaluate computational transcendence on this framework. 4) We demonstrate the efficacy of our model specifically in context of adaptability and resilience of autonomous agents using this framework. 5) Finally we show that our model outperforms baseline model of reciprocity.

### 1.1 Related work

Emerging research areas like Artificial Moral Agents (AMA), Reflective Equilibrium (RE), and Value Sensitive Design (VSD) have proposed several paradigms to formally represent moral and responsible behavior from which, multiple insights can be discerned Jobin et al. (2019); Daniels (1979); Floridi and Sanders (2004); Friedman (1996); Friedman et al. (2002); Fossa (2018); Wallach et al. (2008, 2010); Awad et al. (2022); Santoni de Sio and Van den Hoven (2018). There is a continuum of maturity in moral reasoning between machines and humans Fossa (2018); moral considerations are applicable to any agent (natural or artificial) when it satisfies the following characteristics: interactivity, proactive functioning, and adaptability Floridi and Sanders (2004); moral considerations may require reasoning beyond the current level of abstraction at which an agent is operating Floridi and Sanders (2004); Wallach et al. (2010); moral or responsible behavior is a combination of both top-down imposed norms, and universal principles of morality that are embodied by each agent individually Wallach et al. (2008, 2010); Allen et al. (2005). Responsible behavior is also non-monotonic in nature, requiring belief revisions of one’s deeply held beliefs and values when in conflict with externally imposed expected behavior Rawls (2009); Daniels (1979); Jobin et al. (2019). Cooperative games involving the formation of coalitions have also been explored in the context of responsible autonomy Ahmed and Karlapalem (2022). In these cases, agents are already expected to, or are programmed to cooperate, and the main dilemma is the formation of coalitions.

Different forms of computational foundations have also been explored for agent morality Tolmeijer et al. (2020); Dignum (2017). Three paradigms have emerged as significant alternatives: *consequentialism*, *deontology* and *virtue ethics* as summarized in Table 1. Consequentialism focuses on the end result of an action and selects the action which maximizes the utility or well-being for all agents in the system. Deontology on the other hand, focuses on whether the actions are based on specific, widely-accepted rules or norms–the results of actions are irrelevant in this context. Based on Kant’s categorical and practical imperatives Kant (1785), actions should be such that it can become a universal law and it should not treat people as means to an end. Finally virtue ethics focuses on upholding good virtues in the appropriate amount. The focus in this case is not on actions or consequences but on the agent itself and its virtuous character. We propose transcendence as a fourth paradigm, which we posit leads to emergent responsible behaviour due to the elastic sense of self of agents combined with traditional methods of upholding self-interest and utility maximization.

**TABLE 1:** Comparison between different models of ethics and computational transcendence.

	Consequentialism	Deontology	Virtue Ethics	Computational Transcendence
Description	An action is right if it has the best consequences for everyone	An action is right if it is based on a moral rule or principle	An action is right if it is based on good virtues in the right amount usually as demonstrated by virtuous agents	An action tends to be right if one’s sense of self includes other stakeholders
Central Paradigm	Outputs matter not actions or intentions	Correct rules matter, results are irrelevant	Focus on the attributes of the agent	Elastic sense of self that includes the interests of other stakeholders as one’s own

### 1.2 Agency and identity

Autonomous agency is typically modeled as a utility maximization process, given a self-interest function. Self-interest in turn, is represented by a preference relationship among choices that, augmented with a set of beliefs, can lead to intentions and goal-setting by the agent. An agent may also indulge in speculative reasoning about the future, as well as reflective processes, where the agent examines its beliefs and actions, resulting in revision of beliefs and strategies.

While considerable amount of research has focused on formulating strategies for utility maximization, given a specification of self-interest; to the best of our knowledge, there seems to be little research interest in modeling how an agent’s self-interest itself is formed.

Computational foundations of rational choice, come from the work of von Neumann and Morgenstern Morgenstern and Von Neumann (1953), that is also now called the “classical” model. This theory is based on representing self-interest in the form of preference functions between pairs of choices. Ordinal preference relations between pairs of choices are converted to numerical payoffs based on equating expected payoffs of a conflicting set of choices. Classical rational choice theory is widely used in computational modeling of agency. However, the classical model has also received criticism from various quarters about its shortcomings when trying to model *human* agency. In his critique called “Rational Fools” Sen (1977), Sen argues that our autonomy comes from our sense of who we are, and it is too simplistic to reduce this concept to a preference relation between pairs of choices. Specifically, Sen argues that humans display an innate sense of trust and empathy towards others, and assume a basic level of trust to exist even among self-interested strangers. This critique of the classical model lead to the development of the theory of rational empathy and welfare economics.

While we may be far from a comprehensive model of our sense of self, in this work, we focus on a specific characteristic that may hold the key for the innate sense of responsibility and ethics in humans. We call this the “*elastic*” nature of our sense of self.

In humans, our sense of self is rarely confined to the boundaries of our physical being Hanson (2009). We often attach a part of our sense of self to some external entity, which is when we are said to “identify” with that entity. For instance, some external entities that humans typically identify with, include: their family, religion, gender, ethnicity, race, country, and so on.

Identifying with an external entity is characteristically different from a *rational association* with the entity. Rational association is driven by self-interest, and exists only as long as the association serves our self-interest. But when we *identify* with an external entity, it becomes a part of our sense of self, and *its* interests become part of *our* self-interest. The elastic nature of our sense of self, also forms the basis for social identity Jenkins (2014) that builds a sense of belongingness and loyalty towards something bigger than one’s individual self.

Identification with external entities is not uniformly distributed. An agent may experience a “semantic distance” to an external entity, with its identity *attenuating* over distance. For instance, a parent may rejoice in the successes of others’ children belonging to the same community or nationality that the parent identifies with, but would likely value the successes of their own children much more intensely.

We are not commenting on what *ought* to be the “correct” way in which an agent should curate its identity and semantic distance with other entities. Regardless of what is the “correct” distribution of our sense of self, we posit that the elastic nature of our sense of self, is key to the emergence of responsible behavior in a way that is consistent with, and not in conflict with, self-interest considerations.

### 1.3 Computational transcendence

We now take a formal approach to modeling an elastic sense of self, that we call *computational transcendence*. Given an agent *a*, the sense of self of *a* is described as:

$$S\left(a\right)=\left(I_{a},d_{a},\gamma_{a}\right)$$ (1)

Here *I*_*a*_ is the set of *identity objects* that the agent identifies with. Here *a* ∈ *I*_*a*_, which represents a reflection property in that, the agent itself belongs to its own set of identity objects. The set may contain any number of other entities including other agents, representations of agent collections like an ethnic group or a nation; or even abstract concepts, like “human rights,” or “gender pride,” for example. The criteria for any object to be a member of an identity set of some agent is that, it should be possible to represent the object as a *stakeholder* in the system whose interests need to be furthered and protected, with the stakeholder receiving positive or negative payoffs based on the choices made by agents in the system.

The term $d_{a}:\left\{a\right\}\times I\to R^{+}$ represents the “semantic distance” between *a* and some object in its identity set, with *d*_*a*_(*a*) = 0 ordinarily. The term *γ*_*a*_ ∈ [0, 1] represents the transcendence level or the “elasticity” of the agent’s sense of self, indicating how its sense of self attaches to objects in its identity set. Values of *γ*_*a*_ near 0 indicate that the agent’s sense of self is largely inelastic, while values of *γ*_*a*_ near 1 indicate high levels of elasticity. Agent *a*, with elasticity *γ*_*a*_ is said to identify with an object at distance *d* with an attenuation of $\gamma_{a}^{d}$ .

**FIGURE 1:**
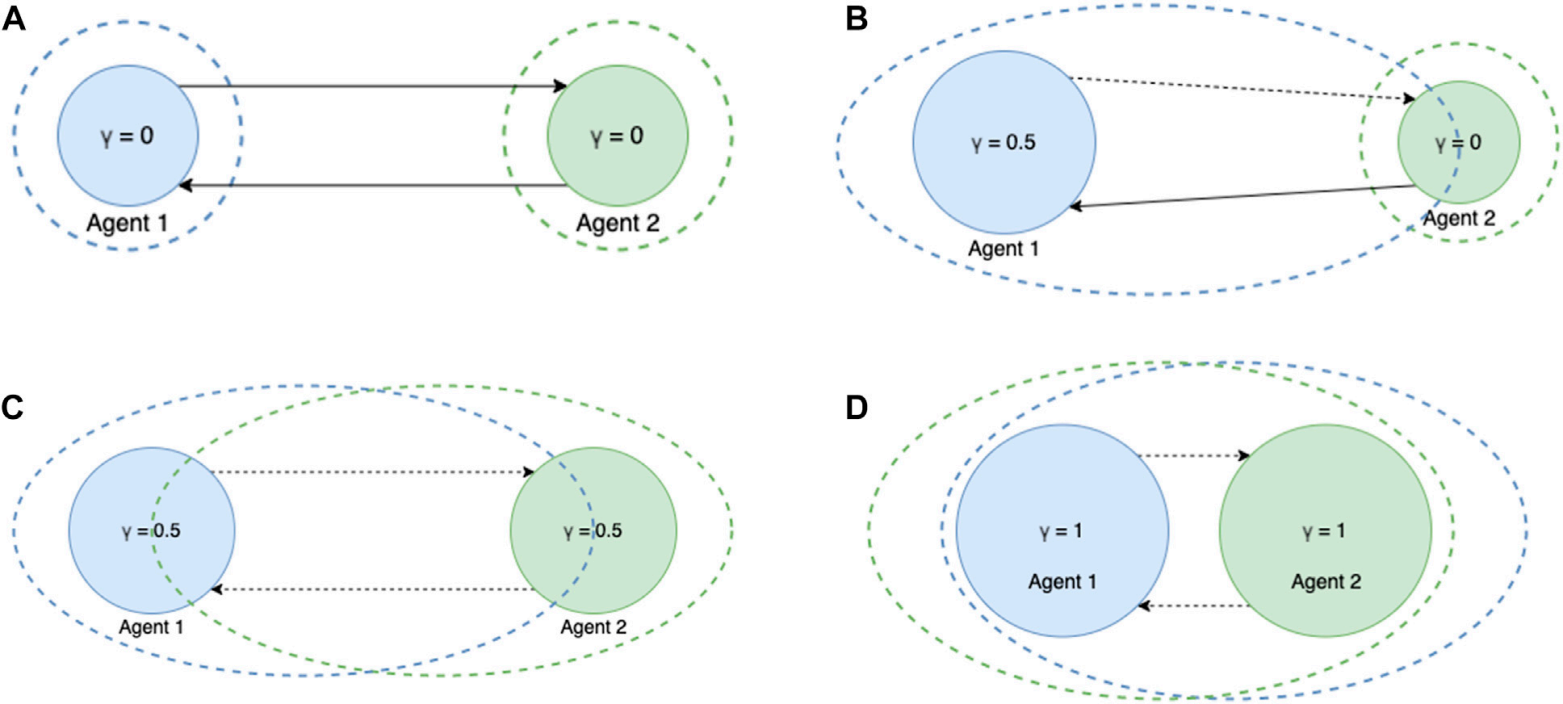
Demonstrating interaction between agents of different transcendence levels **(A)** Non-transcended *vs*. Non-transcended interaction **(B)** Partially-transcended *vs*. Non-transcended interaction **(C)** Partially-transcended *vs*. Partially-transcended interaction **(D)** Fully-transcended *vs*. Fully-transcended interaction.

Figure 1 schematically depicts rational and identity associations between agents with different transcendence levels. Here, diameter of the circle represents transcendence level of the agent. Dashed circle around the agent represents the elastic sense of self of the agent. Solid lines between two agents represents *rational association* while dotted lines between two agents represents *association of identity*. For example Figure 1A shows interaction between two non-transcended agents where their sense of self is limited to just themselves. Figure 1B shows an interaction between a partially transcended agent and a non-transcended agent. We can see that the sense of self of agent 1 includes 50% of agent 2 where as sense of self of agent 2 is limited to just itself. Figure 1C shows interaction between to partially transcended agents and we observe that both of them include the other by 50% in their sense of self. Finally Figure 1D shows an interaction between two completely transcended agents where they completely include the other in their sense of self. Here we have shown interactions between just a pair of agents, however it can be extended to a network with multiple agents as well.

Identifying with external entities affects how an agent’s internal valuation or *utility*, is computed based on external rewards or *payoffs* that may be received by elements of its identity set. For any element *o* ∈ *I*_*a*_, let the term *π*_*i*_(*o*) refer to the payoff obtained by object *o* in game or system state *i*. Given this, the utility derived by agent *a* in system state *i* is computed as follows:

$$\begin{array}{ll} u_{i}\left(a\right) & =\frac{1}{Z}\underset{\forall o\in I}{\sum }\gamma_{a}^{d_{a}\left(o\right)}\pi_{i}\left(o\right)\\ Z & =\underset{\forall o\in I}{\sum }\gamma_{a}^{d_{a}\left(o\right)} \end{array}$$ (2)

The above can be understood as a “unit” of self being attached in different proportions to the objects in the identity set, based on their semantic distance and extent of transcendence. For a rational agent, the distance from an agent to itself is zero, and has the least attenuation in utility. This represents a “rational” model of transcendence of one’s identity towards other objects.

Using this model, we can also construct a model for “selfless” or “altruistic” agents where the semantic distance to itself is *more* than the semantic distance to other objects in its identity set. Such agents value the welfare of other entities more than their own welfare. However in this paper, we restrict our inquiry only to “rational” transcendence, where the semantic distance to oneself is zero.

To illustrate the impact of an elastic sense of identity on responsible actions, consider the game of Prisoners’ Dilemma (PD) as shown in Table 2. The Prisoners’ Dilemma represents a situation where agents have to choose to cooperate (C) or defect (D) on the other. When both agents cooperate, they are rewarded with a payoff *R* (6 in the example). However, as long as one of the agents chooses to cooperate, the other agent has an incentive to defect, and end up with a much higher payoff *T* (10 in the example), at the cost *S* of the cooperating agent (0 in the example). Hence, an agent choosing to cooperate, runs the risk of getting exploited by the other agent. And when both agents choose to defect on the other, they end up in a state of “anarchy” with a much lesser payoff *P* (1 in the example), than had they both chosen to cooperate. The four kinds of payoffs have the following inequality relation: *T* > *R* > *P* > *S*. Here, cooperation is seen as the “responsible” choice, and defection is the “irresponsible” choice. Acting responsibly leads to overall good, but as long as one agent is acting responsibly, there is a rational incentive for the other agent to exploit this, by defecting on the other.

**TABLE 2:** Payoff matrix for 2-player Prisoner’s dilemma.

		Player A	
		C	D
Player B	C	R = 6, R = 6	S = 0, T = 10
	D	T = 10, S = 0	P = 1, P = 1

When played as a one-off transaction, there is no rational incentive for an agent to choose to cooperate. Regardless of whether an agent is known to choose cooperate or defect, it makes rational sense for the other agent to choose *D* over *C*. The state *DD* is the Nash equilibrium, representing the mutual best response by both agents, given the choice of the other. The choice *D* strictly dominates over choice *C*, as regardless of what the other agent chooses, an agent is better off choosing *D* over *C*. The only way agents in a PD game find a rational incentive to cooperate, is when the game is played in an *iterated* manner, with evolutionary adjustments allowing agents to change strategies over time Axelrod and Hamilton (1981); Sadhukhan et al. (2022).

However, with an elastic sense of identity, we can create a rational incentive for the agents to cooperate, even in a one-off transaction. Instead of curating strategies, here we change the agents’ *sense of self*, to include the other agent, to different extents.

Without loss of generality, consider agent *A*, and let the payoff in game state *i* be denoted as *a*_*i*_ (correspondingly, *b*_*i*_ for the other agent). With an elastic identity that includes the other agent in one’s identity set at a distance of *d*, the derived utility of agent *A* in game state *i* is given by (from Eq. 2):

$$u_{i}\left(A\right)=\frac{1}{1+\gamma^{d}}\left[a_{i}+\gamma^{d}b_{i}\right]$$ (3)

The *expected utility* of a choice (either *C* or *D*) is computed by the utility accrued at all possible game states on making this choice, along with the probability of this game state. Since we make no further assumptions, all game states are considered equally probable. Hence, the expected utility for choosing a given choice is computed as follows:

$$\begin{array}{ll} & \;E_{A}\left(C\right)=0.5\cdot u_{CC}\left(A\right)+0.5\cdot u_{CD}\left(A\right)\\ & \;E_{A}\left(D\right)=0.5\cdot u_{DC}\left(A\right)+0.5\cdot u_{DD}\left(A\right) \end{array}$$ (4)

where:

$$u_{CC}\left(A\right)=Ru_{CD}\left(A\right)=\frac{S+\gamma^{d}T}{1+\gamma^{d}}u_{DC}\left(A\right)=\frac{T+\gamma^{d}S}{1+\gamma^{d}}u_{DD}\left(A\right)=P$$ (5)

**FIGURE 2:**
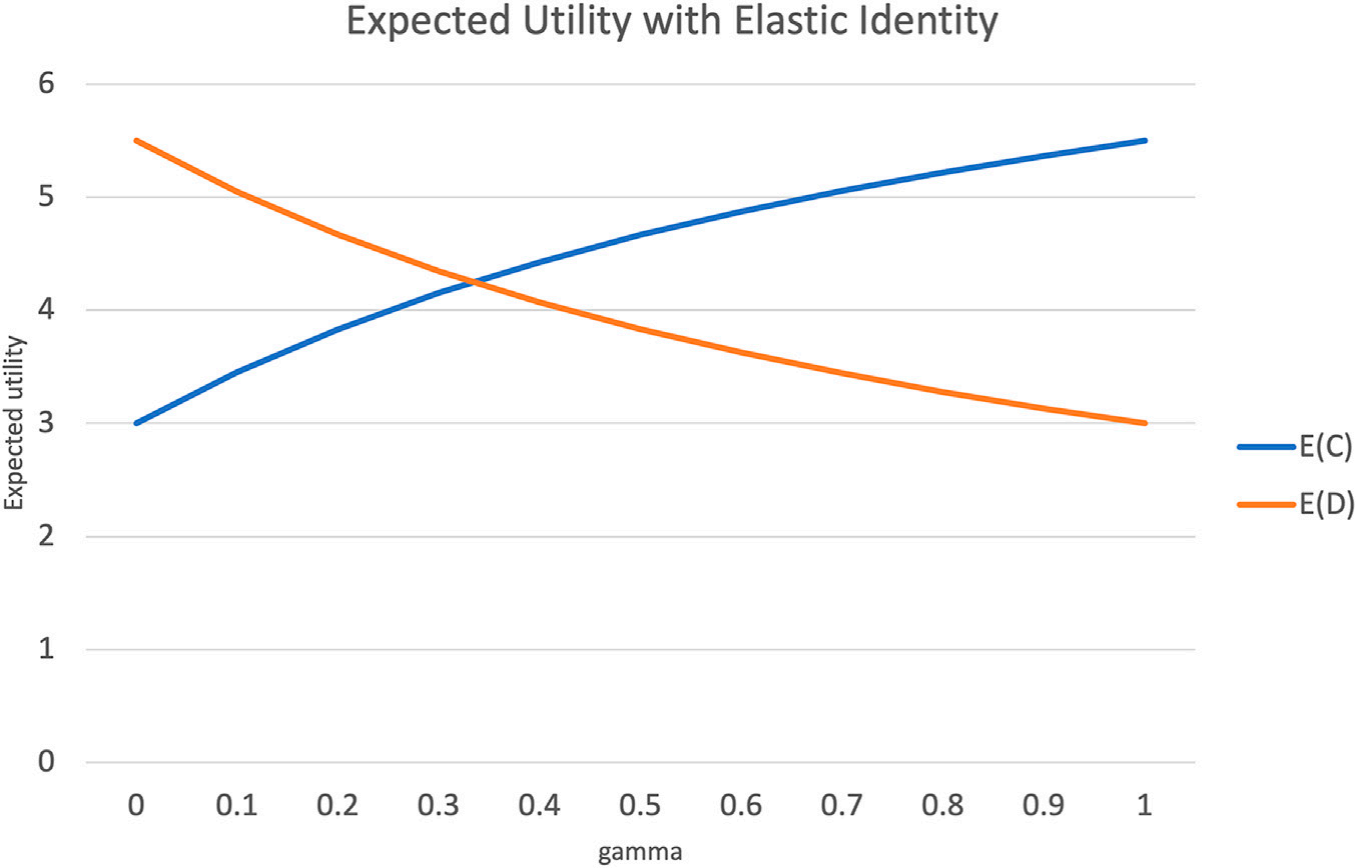
Change in expected utility with increased elasticity of sense of self, and semantic distance *d* = 1.

Figure 2 plots the expected utility from choosing *C* or *D* over varying values of *γ* or the elasticity in one’s sense of self, for a semantic distance *d* = 1.

When *γ* = 0, this becomes the usual PD game, where the expected utility from choosing *D* is much higher than the expected utility from choosing *C*. However, as *γ* increases, with agent *A* identifying more and more with agent *B*, the expected utility of choosing *C* overtakes that of choosing *D*. When *γ* = 1 the sense of self is evenly split between an agent and the other. In this state, the PD game effectively “flips over” with *C* and *D* swapping places with respect to expected utility. Such a state is also called *rational altruism* Khalil (2004); Andreoni and Miller (2008). In this state, it makes as much rational sense to choose to cooperate, as it made rational sense in the conventional PD to choose to defect on the other.

An important caveat is in order here. Suppose agent *A* maximally identifies with the other agent (*γ* = 1), which gives it a rational incentive to offer cooperation. Whether cooperation will actually emerge, will depend on how much the other agent identifies with *A*. Suppose that the other player doesn’t identify with *A*, and chooses to defect instead. In this case, the other agent obtains a payoff of 10 (which is also its internal utility), while agent *A* still gets an internal *utility* of 5 according to Eq. 3, even though its extrinsic payoff is zero. This suggests that a transcended agent that identifies with the other, “feels” gratified with the other agent’s success, even if it comes at the cost of one’s own prospects! In a later section we will see how this “feeling gratified” by other’s success affects the resilience of a population of transcended agents.

Ensuring a minimum extent of identity transcendence across the population, is a major challenge with human societies. However, with engineered AI systems, it is possible for the system designer to *mandate* a minimum level of transcendence for all agents in the system. We will address this issue of engineering AI systems using computational transcendence, in the next section. We also address potential hurdles towards trivially making all agents transcend to the fullest extent possible, to create a population of rational altruists.

Table 3 shows another example game that extends the PD over three players (we shall be using the terms “players” and “agents” interchangeably). This game comprises of three players *A*, *B* and *C*, having to choose between two choices, namely *H* and *T*. The payoff sequence shown in each cell are respectively that of players *A*, *B* and *C* in that order. All three players independently choose their choices.

**TABLE 3:** Payoff Matrix for 3-player collusion game. Payoffs are in the order (*π*_*A*_, *π*_*B*_, *π*_*C*_).

		Players A and B			
		HH	HT	TH	TT
Player C	H	6,6,6	6,1,6	1,6,6	10,10,2
	T	6,6,1	2,10,10	10,2,10	0,0,0

The state *HHH*, where all players choose *H*, results in a fairly high and equitable distribution of payoffs. This state is also the Nash equilibrium, since there is no incentive for any player to unilaterally change its choice, to obtain a better payoff, as long as the other players remain in this choice. Also, since all players get the same payoff in this state, there is no conflict among the players.

However, the game admits the formation of a *collusion*, where any two players can collude and choose *T*, and get a better payoff for themselves at the cost of the third player, as long as the third player remains at *H*. However, when all three players choose *T*, the game collapses with a zero payoff for everyone. Individually, choosing *H* is the dominant strategy, however with collusion, two players decide to choose *T* together at the cost of third player. As before, choosing *H* is the responsible choice that brings overall benefit to the population, but runs the risk of getting exploited, as a result of other players colluding to act irresponsibly.

When combined with computational transcendence, this game not only illustrates the dynamics of collusion, but also the dynamics of social identities at different granularity levels. Societies often encourage responsible behavior from its individuals, by forming communities and promoting a sense of collective identity. However, when large populations contain several communities that compete for identification from individuals, this often results in a contention between one’s communal identity and the global identity of the population Deshmukh et al. (2021).

In this example, individuals transcend with an elasticity *γ*, to identify either with the *world* comprising of all three players, or the *community* or *group* identity formed by the other player with the same choice. The semantic distance that one has with the world or the group, may vary. Agents may identify themselves firstly as citizens of the *world*, and then as members of their *community*; or vice versa. We use the following possible semantic distances *d* ∈ {1, 2, *∞*} with which, an individual identifies with the group or the world. If the individual does not identify with the group or the world, its distance is set to *∞*.

Let *d*_*g*_ and *d*_*w*_ be the semantic distance from a given agent *a* to the group or the world, and let *π*_*g*_ and *π*_*w*_ represent the payoffs of the group and the world, respectively. Let *π*_*a*_ represent the payoff for player *a*. The transcended utility for the agent *a* in game state *i* is given by (Equation 2):

$$u_{a}\left(i\right)=\frac{1}{1+\gamma^{d_{g}}+\gamma^{d_{w}}}\left[\pi_{a}\left(i\right)+\gamma^{d_{g}}\pi_{g}\left(i\right)+\gamma^{d_{w}}\pi_{w}\left(i\right)\right]$$ (6)

For different combinations of distances, we now plot the expected utility from choosing *H* or *T* for any given player.

**FIGURE 3:**
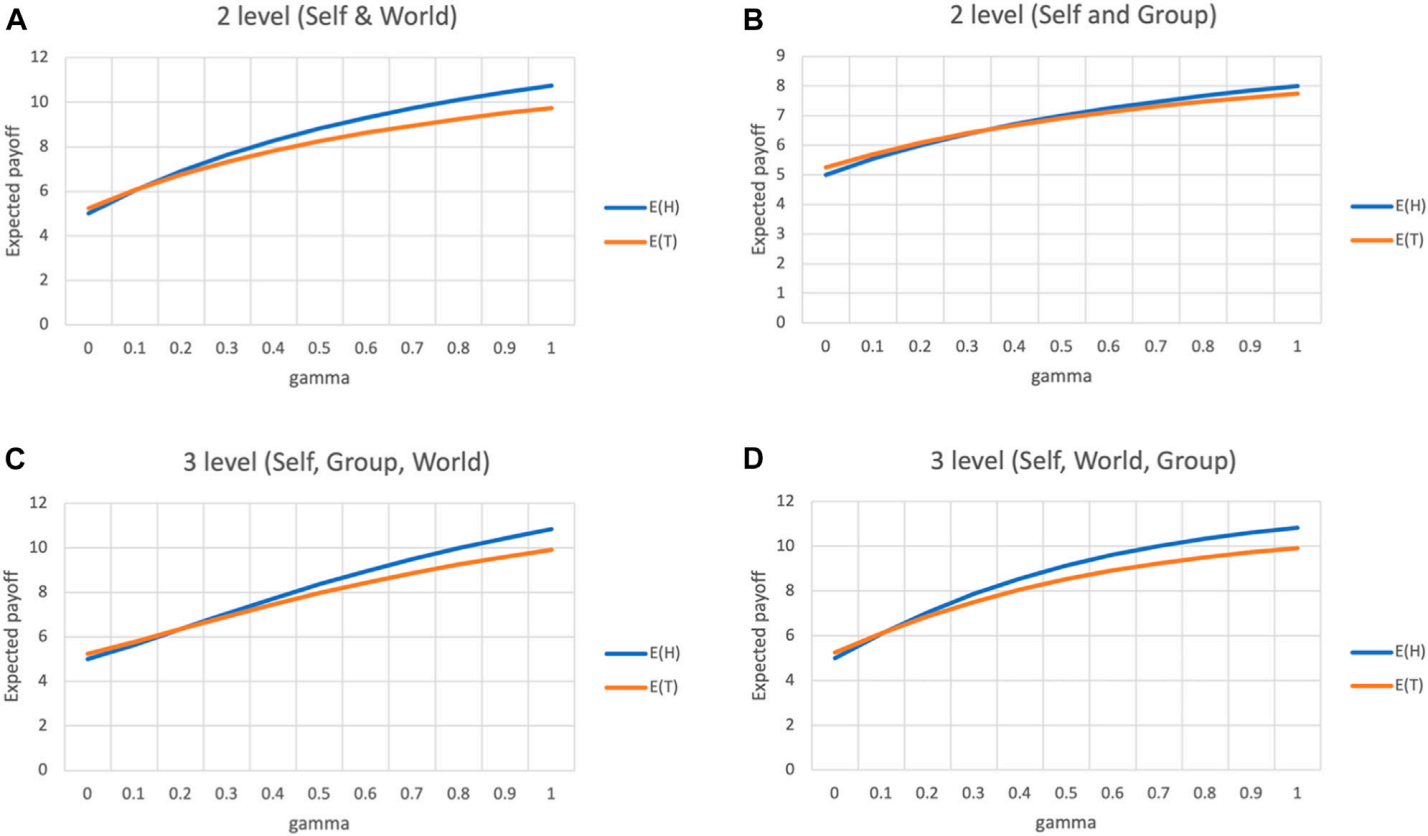
Transcendence in a three player PD game **(A)** Transcendence from Self to World **(B)** Transcendence from Self to Group **(C)** Transcendence from Self to Group (*d*_*g*_ = 1) to World (*d*_*w*_ = 2) **(D)** Transcendence from Self to Group (*d*_*g*_ = 2) to World (*d*_*w*_ = 1).

Figures 3A,B plot the expected utility of choosing *H* and *T* for a player, that transcends to identify with either the world or the group, respectively. Here, an agent identifies with either the world or the group, which means either *d*_*g*_ or *d*_*w*_ is set to 1, and the other term set to *∞*. When *γ* = 0 the expected utility from choosing *T* is slightly higher than that of choosing *H* (5.25 and 5 respectively). However, as *γ* increases, the expected utility of *H* overtakes that of *T*. Regardless of whether an agent identifies with the group or the world, the higher the extent of transcendence, the more rationale there is to choose the more responsible choice. We can also see that identifying with the world makes the utility of *H* grow faster than that of *T*, as compared to identifying with the group.

Figures 3C,D depict a three-stage transcendence, where an agent identifies with both the group and the world, to different extents. In the first case, the agent identifies with the group and the world at a semantic distance of 1 and 2 respectively, while the distances are reversed in the second case.

In both cases, *H* overtakes *T* and reach the same difference (10.83 and 9.91 respectively), when *γ* = 1. And although the intermediate values for *E*(*H*) and *E*(*T*) differ between the two cases, the overall trapezoid area under curve (AUC) is exactly the same for both cases (*AUC*(*E*(*H*)) = 0.292, *AUC*(*E*(*T*)) = 0.233). This suggests that as long as the world or the entire collective is part of the identity set of an agent, it does not matter to what extent the agent identifies with its community.

We will need to clarify here, why choice *H* is considered a “responsible” choice, while *T* is considered an “irresponsible” choice. With the earlier example of a 2-player PD (Table 2) it is more clear. The game state *CC* has the highest collective payoff and the minimum disparity among the payoffs. With the 3-player collusion game (Table 3), the state *HHH* does have the least disparity in payoffs, but is not the state with the highest collective payoff. The collusive state *HTT* for example, yields a collective payoff of 22, while *HHH* yields a collective payoff of 18. With a simple utilitarian model that considers the sum of individual payoffs as collective good, *HTT* appears better for the collective than *HHH*. But this does not factor the disparity in payoffs.

A better model for assessing outcomes from responsible choices is the Nash bargaining metric Binmore et al. (1986). The Nash bargaining metric computes collective extrinsic utility as the product of individual payoffs. In games with non-negative payoffs, this metric is shown to account for both maximization of gains and minimization of disparity in utilities between the players.

With the Nash bargaining metric *HTT* has an overall payoff of 200, while *HHH* outperforms this with an overall payoff of 216. Similarly, in the case of 2-player PD, an exploitative game state (*CD* or *DC*) has a Nash bargaining payoff of 0, while the cooperative game state has a value of 36.

Extending one’s sense of self beyond one own individual seems to provide a natural incentive for acting responsibly. In the next sections, we build a generic multi-agent framework to implement transcendence, and evaluate various scenarios involving responsible behavior.

## 2 Methodology

With the basic model of transcendence presented so far, we now turn to the question of how can we engineer multi-agent AI systems using computational transcendence. For this, we propose a generic multi-agent framework that incorporates transcendence in autonomous decision-making.

We consider a community of autonomous agents forming a network represented by a simple, undirected graph, with the nodes representing agents, and edges representing pair-wise interactions between agents.

Each agent performs two classes of activities: (a). Initiating network traffic, and (b). Forwarding of network traffic from others. When agents forward network traffic from others, they are said to be *intermediaries*. Intermediaries face two classes of choices: a *responsible* choice that may incur a cost, but bring overall benefit to the network; and a *selfish* or *irresponsible* choice that minimizes cost to the agent, but may negatively affect overall network performance.

When an agent initiates network traffic, it gets a payoff when the packet successfully reaches its destination. If the packet gets dropped midway, the initiator incurs a net cost. In contrast, when an agent forwards a packet from another agent, it incurs a cost in performing this operation, but does not obtain any payoff. Given this, there is no ordinary incentive for an agent to forward packets sent by others, while agents initiating network traffic need the cooperation of intermediaries to successfully complete their task.

Formally, the multi-agent framework is represented as follows:

$$G=\left(A,E,\gamma ,d\right)$$ (7)

Here, *A* is the set of agents, *E* ⊆ *A* ^2^ is the set of pairwise interaction among agents, *γ* is the elasticity or the transcendence level that is set for the community as a whole, and $d:A\times A\to R^{+}$ is the set of non-negative, semantic distances between agents. Note that semantic distance is a perceived logical distance, and need not be symmetric between pairs of agents.

Let *i* ∈ *V* be any agent in the network, and let Γ(*i*) denote the set of other agents with which it is interacting. For any *s* ∈ Γ(*i*), the term *d*(*i*, *s*) represents the semantic distance perceived by *i* towards *s*, the term *utility*(*i*, *s*) represents the total utility accrued by *i* with its interaction with *s*, and the term *cost*(*i*, *s*) represents the total cost incurred by *i* from its interaction with *s*. Analogous terms are defined from the perspective of *s* towards *i*.

When *i* receives a packet from *s*, it becomes the *intermediary*, and *s* becomes the *source*. The intermediary has two choices: forward (f), or drop (d). The choice made by *i* affects not only its own utility, but also the utility of the source *s*, as shown in Table 4.

**TABLE 4:** Utilities and Costs for intermediary agent (i) and source agent (s) as a result of decisions taken by intermediary.

	Utility (u)	Agent i	Agent s		Cost (c)	Agent i	Agent s
	Forward (f)	*γ* ^*d*(*i*,*s*)^ *μ*	*μ*		Forward (f)	*κ*_*i*_	0
Agent i				Agent i			
	Drop (d)	− *γ* ^*d*(*i*,*s*)^ *μ*	− *μ*		Drop (d)	0	0

In the payoff matrix from Table 4, *μ* is the extrinsic utility or payoff received by the source node, for successful delivery of a packet; and *κ*_*i*_ is the cost incurred by the intermediary for forwarding of a packet. When agent *i* forwards the packet sent by agent *s*, agent *i* incurs a cost of *κ*_*i*_, and agent *s* gets a payoff of *μ*. But since agent *i* identifies with *s*, it too gets a utility of *γ* ^*d*(*i*,*s*)^ *μ*. Similarly, when agent *i* drops the packet, the source agent *s* incurs a loss *μ*, which is also shared by agent *i* due to transcendence, deprecated by a factor *γ* ^*d*(*i*,*s*)^.

The term *κ*_*i*_ depends not only on the cost of forwarding a packet, but also on other loads on the agent, like battery power in case of mobile agents, and interactions happening on other channels. To account for all this, *κ*_*i*_ is derived from the base packet forwarding cost *κ* as is defined as follows:

$$\kappa_{i}=\kappa +\delta \underset{\forall s\in \Gamma \left(i\right)}{\sum }\left(cost\left(i,s\right)-utility\left(i,s\right)\right)$$ (8)

Here, *κ* is the base operational cost of forwarding a packet, and 0 ≤ *δ* ≤ 1 is a dampening factor that accounts for other costs being borne by the agent. The initiator agent *s* incurs the base cost of *κ* at sending time, while the intermediary *i* incurs a modulated cost *κ*_*i*_ that factors other costs that the agent has to bear, while forwarding the packet.

$$\begin{array}{cc} E\left(f\right) & =\frac{1}{1+\gamma^{d\left(i,j\right)}}\left(-\kappa_{i}+\gamma^{d\left(i,j\right)}*msgUtility\right)\\ E\left(d\right) & =\frac{1}{1+\gamma^{d\left(i,j\right)}}\left(-\gamma^{d\left(i,j\right)}*msgUtility\right) \end{array}$$ (9)

Eq. 9 shows the expected payoff of forwarding (f) and dropping (d) of a packet from the intermediary *i* to the next agent *j*. Based on these expected utilities, the agent *i* decides whether to forward or drop the packet, by converting expected utilities to probabilities, using a softmax function as follows:

$$\begin{array}{c} prob\left(f\right)=\frac{e^{E\left(f\right)}}{e^{E\left(f\right)}+e^{E\left(d\right)}}\\ prob\left(d\right)=\frac{e^{E\left(d\right)}}{e^{E\left(f\right)}+e^{E\left(d\right)}} \end{array}$$ (10)

If the packet is forwarded, *i* adds its utility obtained by it (*γ* ^*d*(*i*,*s*)^ *μ*) to *utility*(*i*, *s*), and the base operational cost incurred by it (*κ*) to *cost*(*i*, *s*), while the source agent *s* adds the utility obtained by it (*μ*) to *utility*(*s*, *i*). Similarly, if agent *i* drops the packet, a loss of -*γ* ^*d*(*i*,*s*)^ *μ* and − *μ* are recorded for *utility*(*i*, *s*) and *utility*(*s*, *i*) respectively.

## 3 Experiments

The framework introduced above is used to perform three stages of experiments involving transcendence. In the first stage, we build a network of agents where all of them have been programmed with a given value of transcendence (*γ*) and respond to externalities by adjusting their semantic distances between one another; in the second stage, agents discover their optimal level of transcendence with respect to their environment, by making evolutionary adaptations; and in the third stage, a network of agents with elastic identity is infused with a population of adversaries which do not transcend, in order to test its resilience.

**FIGURE 4:**
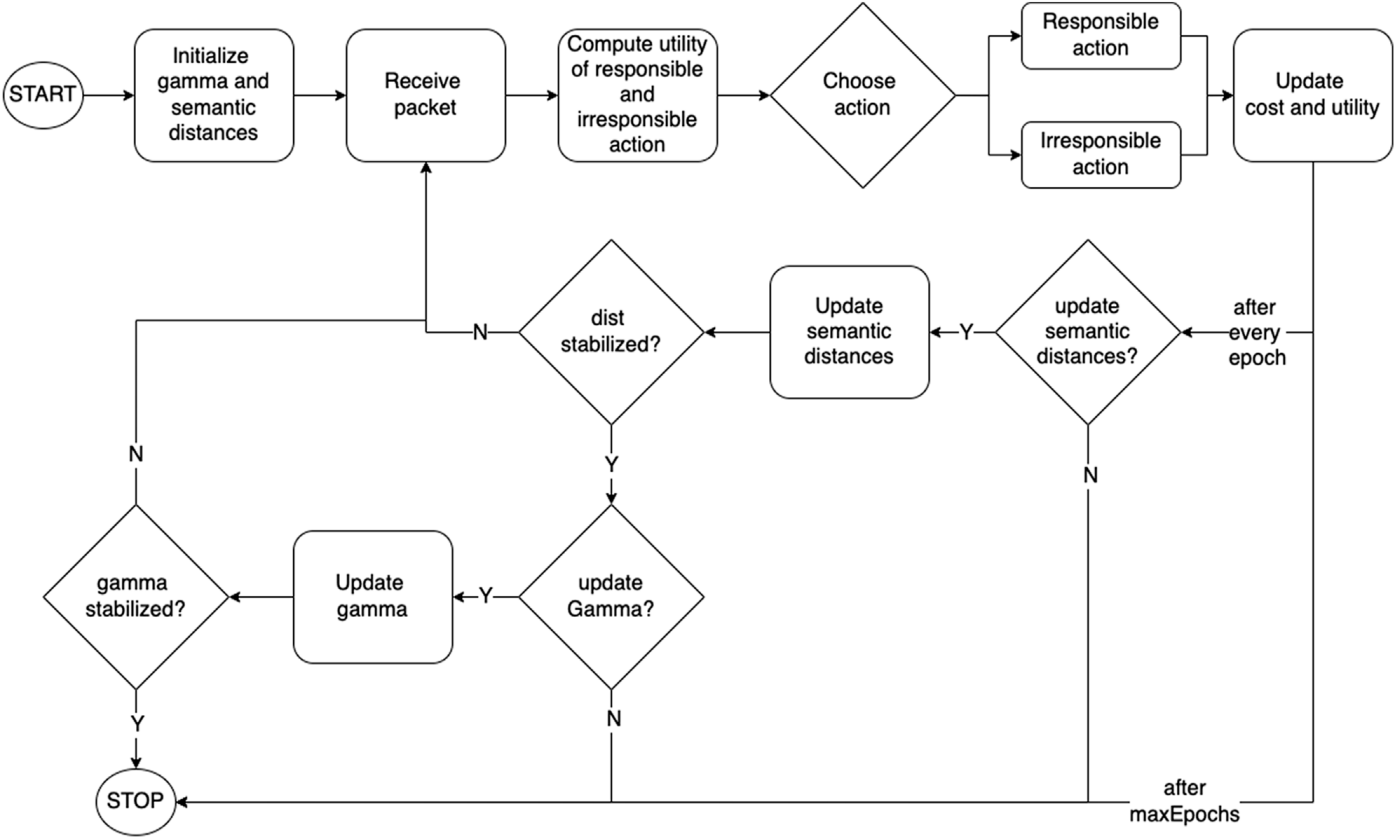
Block Diagram of a Transcended agent.

The first two stages constitute the life cycle of a transcended agent, and is schematically depicted in Figure 4.

### 3.1 Basic network: Curating semantic distances

In the description of the multi-agent framework from the previous section, we can see that when an intermediary chooses to act responsibly, the costs incurred by it are *real*–involving actual expending of energy and resources; but the utility received by it is “virtual”– obtained by *identifying* with the other agent. However, experiencing this virtual utility is crucial, as it results in real benefits for the sender and for the network as a whole, thus leading to responsible autonomy.

The real nature of the costs incurred as opposed to the virtual nature of the benefits accrued, presents a barrier for an agent from naïvely identifying to the maximum extent, with everybody in its neighborhood. Too high a level of transcendence also makes an agent vulnerable for exploitation by other rational agents. In order to practice transcendence in a rational manner, agents employ a defense mechanism by adjusting the semantic distance *d* that they perceive with every other agent in their identity set. This is implemented by interpreting utility and cost on each edge as a reinforcement signal and responding accordingly with distance adjustments.

The framework functions in batches of operations called *epochs*. Each epoch starts with a distribution of semantic distances for each edge, and comprises of a certain number of packets being transmitted across the network, and agents accumulating utilities and costs. The initial distribution of distances may be based on some form of physical or logical separation, specific to the application.

At the end of every epoch, semantic distances between agents are updated based on accumulated utility. Each agent *i* updates its semantic distance to its neighbor *s* as follows:

$$\begin{array}{ll} \Delta d\left(i,s\right) & =\frac{e^{utility\left(i,s\right)}}{\underset{\forall s\in \Gamma \left(i\right)}{\sum }e^{\left|utility\left(i,s\right)\right|}}-\frac{e^{cost\left(i,s\right)}}{\underset{\forall s\in \Gamma \left(i\right)}{\sum }e^{\left|cost\left(i,s\right)\right|}}\\ d_{t}\left(i,s\right) & =d_{t-1}\left(i,s\right)-\lambda \Delta d\left(i,s\right) \end{array}$$ (11)

Here, each agent *i* computes a marginal utility accrued from a specific neighbor *s* as a softmax function that normalizes its utility with overall utility obtained, as well as its cost accrued with *s*, with overall costs accrued from all neighbors. This marginal utility is then scaled with a learning rate *λ* and is used to update the distance between agents *i* and *s*.

### 3.2 Discovering elasticity

In the next stage of the framework development, rather than having a fixed level of transcendence across the network, agents adjust the elasticity (*γ*) of their identity, based on their experience, to balance between the benefits and costs of transcendence. This obviates the need for the system designer to impose a particular transcendence level for all the nodes in the network.

This evolutionary network starts like the basic network model with a given initial value of *γ* and updates its distances. Once the distances stabilize as discussed in the basic network model, the agents update their transcendence levels as follows:

Agents compare the total utility they have *received* from their interactions with other agents (*u*_*R*_), to the total utility they have *given* to other agents in the network (*u*_*G*_). Based on these two parameters, agents update their transcendence levels as follows:

$$\begin{array}{cc} u_{R}\left(a\right) & =\underset{\forall i\in \Gamma \left(a\right)}{\sum }utility\left(a,i\right)-cost\left(a,i\right)\\ u_{G}\left(a\right) & =\underset{\forall i\in \Gamma \left(a\right)}{\sum }utility\left(i,a\right)-cost\left(i,a\right)\\ \Delta \gamma \left(a\right) & =\frac{1}{1+e^{-\left(u_{R}\left(a\right)-u_{G}\left(a\right)\right)}}\\ \gamma \left(a\right) & =\gamma \left(a\right)+\lambda_{g}\Delta \gamma \left(a\right) \end{array}$$ (12)

The rationale here is that, agents are assumed to feel gratified and be more inclined to identify with their environment, when their environment is more generous than their expectations. However, this gratification may be subject to diminishing valuation of returns, modeled by the sigmoid function. The output of the sigmoid funtion, Δ*γ*(*a*) is used as a reinforcement signal by the agent to adjust its elasticity *γ*, scaled by learning rate, *λ*_*g*_. This process is repeated till *γ* updates between consecutive epochs fall below a threshold.

### 3.3 Network resilience

Earlier, we had noted that even with simple, 2-player games, transcendence can lead to cooperation only if both players were to transcend, and identify with the other. If one of them transcends and acts responsibly, it makes even more rational sense for the non-transcended other player to act irresponsibly. Thus, an agent unilaterally deciding to transcend, runs the same risk as that of an agent unilaterally deciding to cooperate in a rational standoff. However, transcendence has subtle differences from a simple rational standoff, as the transcended agent would still derive some *virtual payoff* from the other agent’s gains–which under certain conditions could still be sufficient incentive to keep the population functioning, and to eventually isolate the adversaries based on distance updates.

Given this, in the third stage of the framework design, we ask the question of how *resilient* is a network of transcended agents. A network of agents is said to be resilient, if it cannot be “invaded” by a small population of *adversaries* who can exploit the network’s cooperative nature, to an extent that there is no rational incentive for cooperation anymore.

In order to model this, we introduce *adversarial* agents into a network of transcended agents that act selfishly, and do not display elastic identities. Adversaries are also modeled as rational maximizers even in positioning themselves in the network–occupying network positions of high influence, indicated by degree centrality. We then vary the proportion of adversaries to evaluate the resultant network for multiple metrics.

### 3.4 Baseline comparison

In order to compare how computational transcendence performs as a model for responsible autonomy, we compare it the paradigm of *reciprocity* Gouldner (1960), which is one of the commonly used paradigm for ethics. Reciprocity is based on the logic that an individual’s sense of responsibility is a normative construct that is learned based on observing how other individuals behave in their vicinity. Each agent observes other agents in its neighborhood to learn the rates at which they take up responsibility, or act selfishly, and adopt similar rates for themselves. We compare the basic network of computational transcendence as discussed in Subsection 3.1 with such a model of reciprocity.

## 4 Results

We implement a simulation of the proposed framework, created from a random graph generated using the Erdös-Renyi model Newman (2003) over 100 nodes. A set of 200 packets is generated per epoch, where the start, intermediate, and destination nodes are chosen uniformly at random. The utility *μ* of a packet being successfully delivered, is set to 10 units, and the base operational cost of transmitting packets (*κ*) is set to one-tenth of the utility.

### 4.1 Basic network: Curating attenuation rates

After each epoch, distances are updated by agents based on the marginal utility obtained from its neighbors, and the learning is stopped when changes in distances fall below a threshold *ϵ* set to 0.01. The hyper-parameters *δ* (discount factor for modulating intermediary cost), and *λ* (learning rate), are both set to 0.01. In the simulation runs, the initial set of semantic distances were set to 1 for all the edges, and the simulation was run by varying *γ* from 0.1 to 1.0 with a step size of 0.1.

**FIGURE 5:**
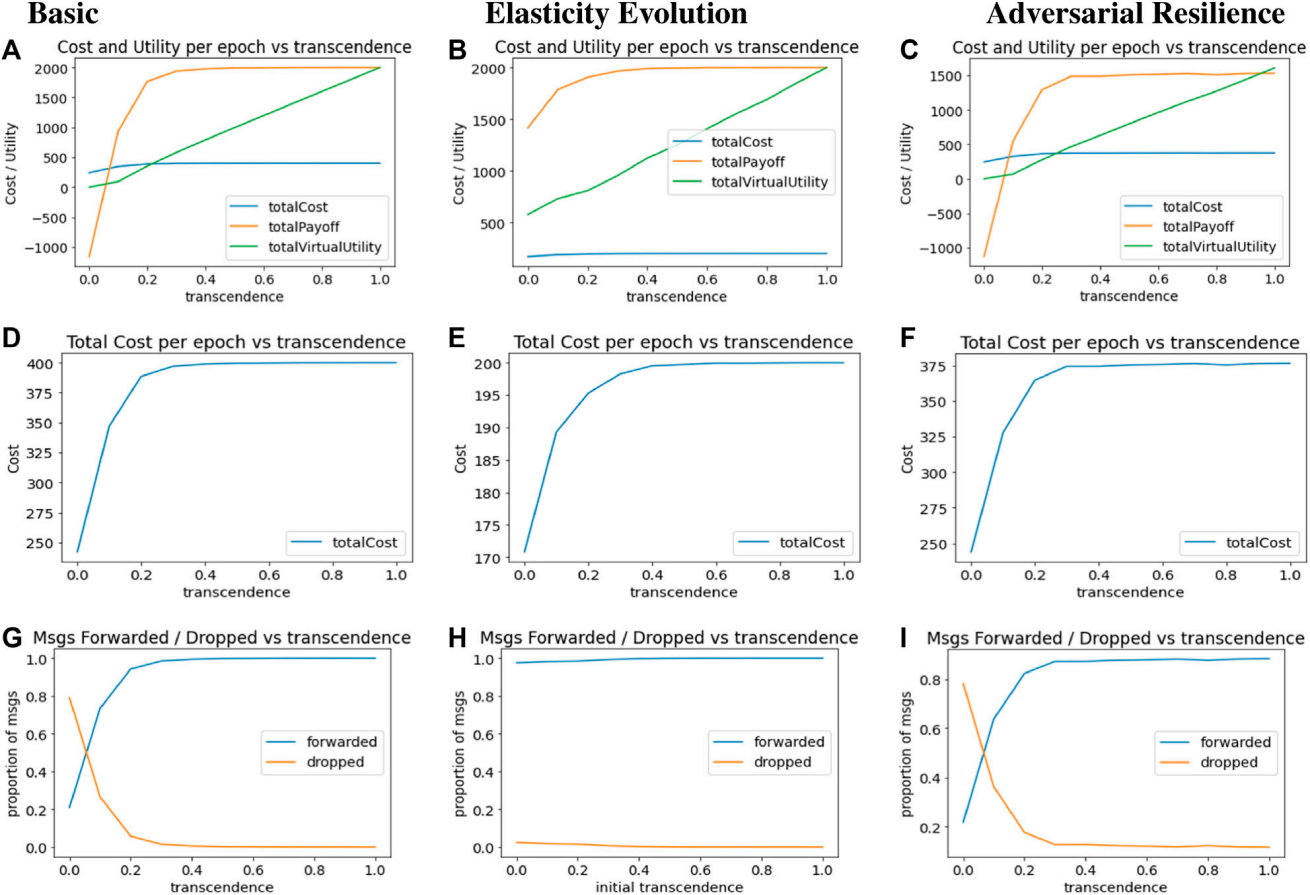
Result Plots for Computational Transcendence in Basic, Evolutionary and Adversarial Networks **(A)** Basic: Total Metrics **(B)** Evolutionary: Total Metrics **(C)** Adversarial: Total Metrics **(D)** Basic: Cost per epoch **(E)** Evolutionary: Cost per epoch **(F)** Adversarial: Cost per epoch **(G)** Basic: Forwarded Ratio **(H)** Evolutionary: Forwarded Ratio **(I)** Adversarial: Forwarded Ratio.

Increase in utility overshadows the increase in cost as *γ* increases, even though at lower levels of *γ*, collective utility is negative. This is shown in Figure 5A. Both virtual utility (accrued by the intermediate agents identifying with the source) and real utility (accrued by the source nodes) increase with higher transcendence.

Figure 5D plots the total cost per epoch against increasing transcendence. While increasing extents of identification with others makes it more attractive to act responsibly, this responsibility also comes with increased costs of operations. Cost of operations increases rapidly with increasing transcendence, and saturates at a point where packet forwards reach 100%.

Figure 5G plots the proportion of packets forwarded and dropped by intermediaries per epoch, with increasing values of elasticity *γ*. Without transcendence (*γ* = 0), there is no incentive for any intermediary to forward packets for others, and packet drops outnumber packet forwards. However, with increasing transcendence, packet forwarding rate rises rapidly, with a similar rapid decline in packet dropping rate.

### 4.2 Discovering elasticity

In second stage of the framework, the system runs in two levels of epochs. At the basic level, distances are updated until they stabilize. This then triggers *γ* updates, where following each such update, distances are updated again till they stabilize. The results are plotted after both distance and *γ* updates stabilize.

The second column in Figure 5, plots the results for the network with evolutionary *γ* updates, that is configured similarly to the basic network model. In these runs, the network was initialized with an common initial value of *γ* for all agents, which forms the *x*-axis. The network then goes through several epochs of distance and *γ* updates until it stabilizes. The *x*-axis shows the initial value of *γ* with which the network was updated, and values in the *y*-axis represents values of corresponding metrics after both distance and *γ* updates have stabilized.

Figure 5B plots cost, utility and virtual utility per epoch for different initial values of *γ* on the *x*-axis. We note that utility and virtual utility levels are higher even for lower initial values of *γ* since the system evolves and eventually reaches a point where agents have a higher level of transcendence and thus get higher utilities–both real and virtual.

Cost trends in Figure 5E are similar to other cases–the cost incurred by agents increases as their initial elasticity levels increase–posing a rational barrier for agents to naïvely identify with others to the fullest extent.

Figure 5H shows that it does not matter what is the initial transcendence level of the agents, the agents are able to reach the stable state of almost 100% forwarded ratio, by the time *γ* updates stabilize. Thus, the algorithm for evolutionary updates obviates the need for the system designer to identify the optimal value of transcendence.

**FIGURE 6:**
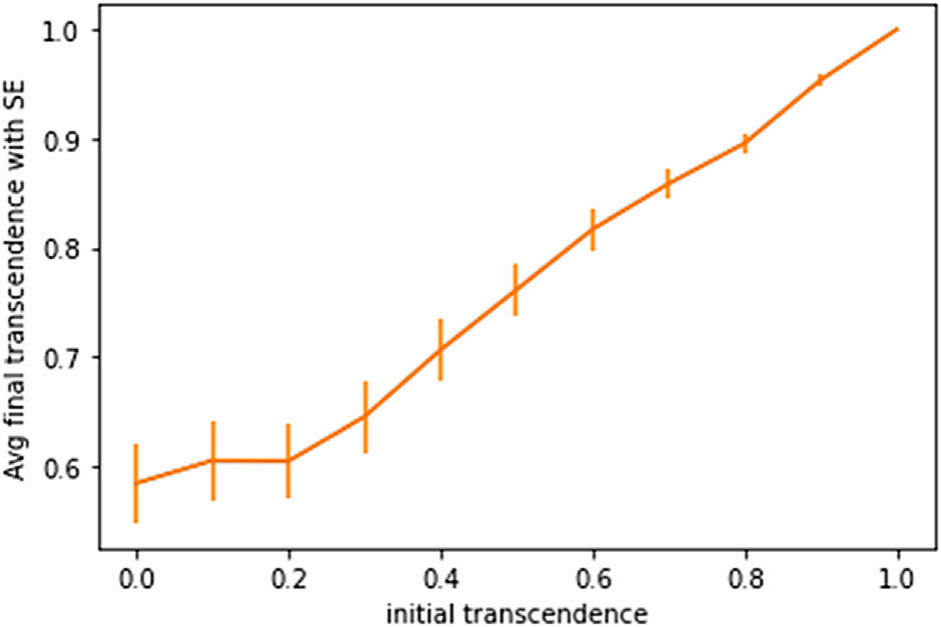
Average value of gamma at equilibrium given initial value of gamma with standard error bars.

Figure 6 shows the average value of *γ* of all the nodes in the graph when *γ* updates stabilize. Here, error bars represent standard error. It shows that for low initial values of *γ*, the system settles to an elasticity of around 0.6, with a sizeable disparity of transcendence levels among different nodes; and for higher initial values of *γ*, elasticity settles down to an even higher value for all nodes–with lower variation among the different nodes.

The final value at which *γ* stabilizes, depends on the topology of the network and the neighborhood for each node. Exploring the impact of network topology on transcendence levels is an interesting problem, but beyond the scope of this paper.

### 4.3 Network resilience

For the third stage of the framework design that evaluates network resilience, to begin with, we consider 20% of the agents in a network to be adversaries, occupying nodes with the highest degree. We then fix the value of *γ* for the rest of the agents at different values, and see how they respond to adversaries, with only distance updates.

The third column in Figure 5 shows the main plots of the resultant system with adversaries. Figure 5C shows that the cost, utility and virtual payoff trends per epoch are very similar to the trends of basic network, indicating that the presence of adversaries is not changing the basic nature of transcendence. However, if we look at the exact values on the *y*-axis, we note that the utility levels are lower with adversarial agents than with the basic network. The average cost per epoch in Figure 5F is similar to the basic network. In Figure 5I, it is interesting to note that despite the presence of 20% adversary agents at key locations, the forward and drop ratio is not significantly affected as compared to the basic network.

**FIGURE 7:**
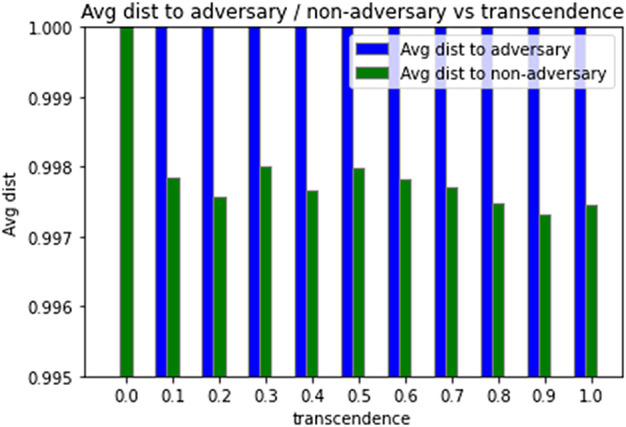
Distance to adversarial and non-adversarial agents.

In the basic network, agents curate their semantic distance (*d*) to their neighbors based on how they were treated by their neighbors. This is the primary defence mechanism that the network has against adversaries as well. Figure 7 shows the average distance of agents to adversary and non-adversary agents for different levels of transcendence (*γ*). For all transcendence levels, we note that when distances stabilize, adversaries are more alienated than non-adversarial agents.

The above empirical results show that the network is resilient in the sense that, it continues to function cooperatively, with more packet forwards than drops, even in the presence of adversaries. In addition, agents distance themselves from the adversaries, thus reducing their degree of membership to transcended agents’ identity set.

**FIGURE 8:**
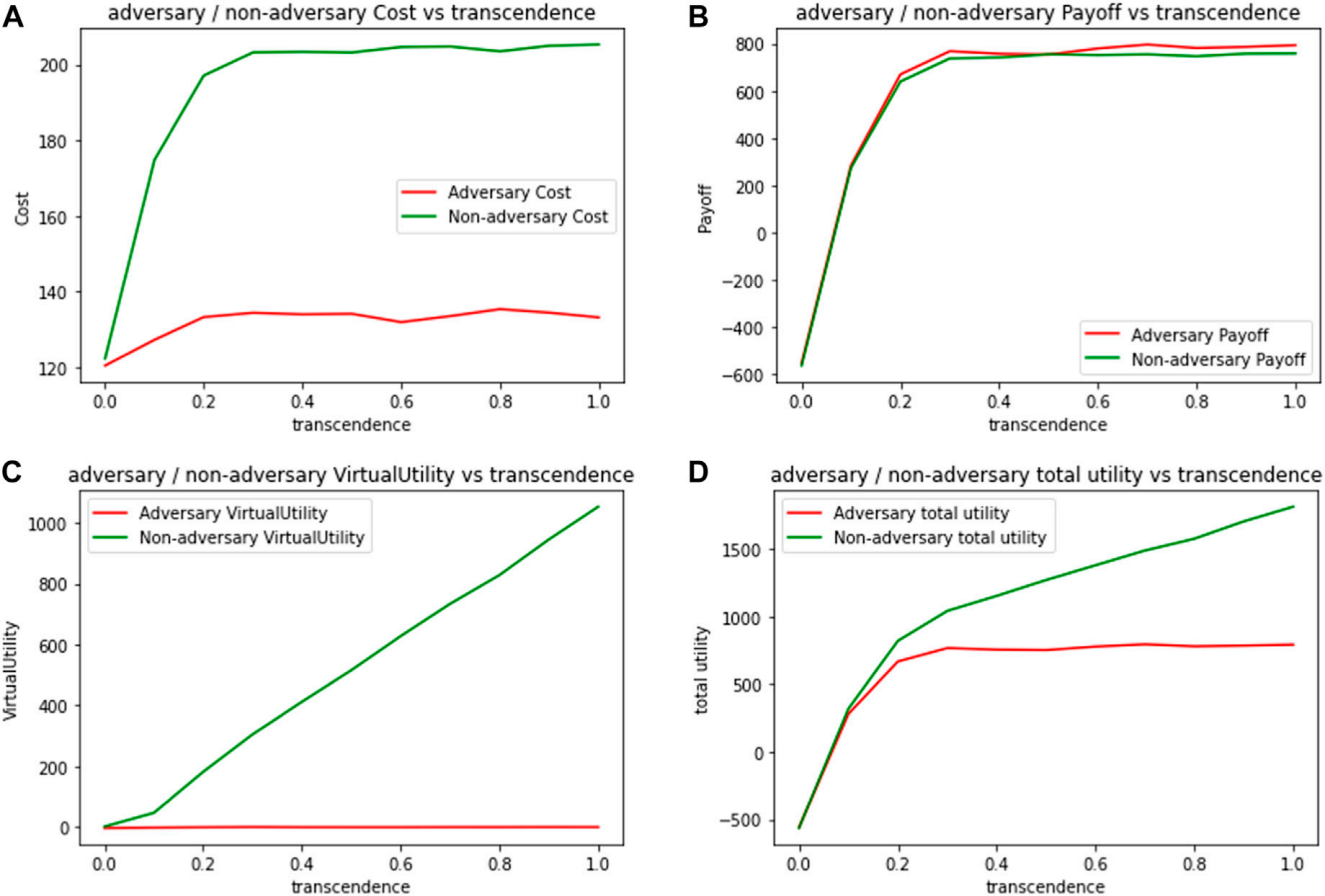
Expected metrics per 100 packets for adversarial and non-adversarial agents by varying transcendence **(A)** Cost **(B)** Payoff **(C)** Virtual Utility **(D)** Total Utility.

It is also worthwhile to also ask how do the adversaries fare in such a network. In Figure 8, we compare cost and utility values for adversary and non-adversary agents. Figure 8A plots the expected cost per 100 packets, for adversary and non-adversary agents when initialized with different levels of transcendence (*γ*). We note that with increasing levels of transcendence, costs for non-adversary agents increase, due to their cooperative behavior as intermediaries. Adversaries only incur costs when initiating a packet, and their expected costs remain almost constant, with increasing transcendence.

Even when we consider real payoffs as shown in Figure 8B, adversaries fare better than non-adversaries since their packets are much more likely to be forwarded. It is only the virtual component of the utility, shown in Figure 8C that provides a rationale for non-adversary agents to continue acting cooperatively. Figure 8D combines real and virtual payoffs for both kinds of agents, and as expected, while the total utility increases for both kinds of agents with increasing transcendence, total utility is a lot higher for the transcended agents because of their virtual component.

This shows some significant nuances about network resilience in the presence of adversaries. Although 20% of the most influential nodes (with highest degree) are adversaries, the network continues to function cooperatively, and derives a high total utility due to transcendence. But, there is no *disincentive* for adversaries to stop them from exploiting the cooperative nature of the network. The only disincentive is the increased semantic distances from other agents, which does not have much impact on their payoffs. Both the network and the adversaries continue to thrive as transcendence levels increase. The virtual component of the utility also ensures that there is no disillusionment for the cooperative players either, that is essential to keep the network functioning.

This brings us to the question of to what extent can such a network accommodate adversaries and continue functioning, and when would it no longer make rational sense for transcended agents to cooperate? We consider this scenario next. We first build a network with a fixed elasticity of transcended agents, set to *γ* = 0.8, and vary the proportion of adversaries. In all the cases, adversaries occupy nodes with the highest degree in the network.

**FIGURE 9:**
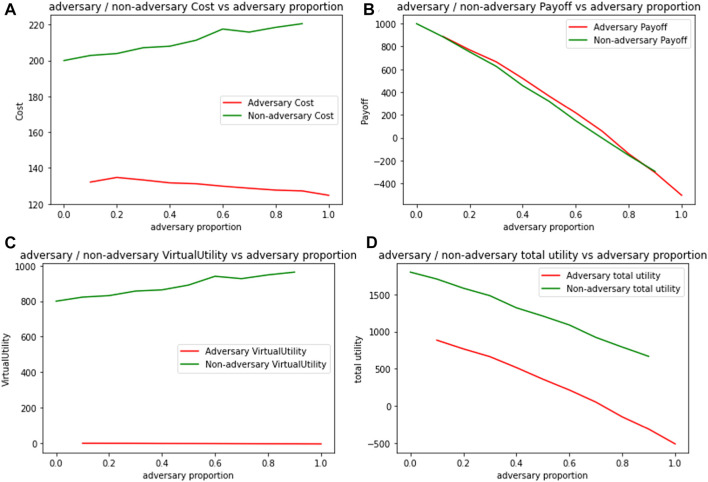
Expected metrics per 100 packets for adversarial and non-adversarial agents by varying adversary proportion **(A)** Cost **(B)** Payoff **(C)** Virtual Utility **(D)** Total Utility.

Figure 9 shows varying trends of cost, payoff, virtual utility and total utility when the proportion of adversaries is varied, as represented on the *x*-axis. Figure 9A shows an increasing cost trend for transcended agents as they try to forward packets in adverse environments and a decreasing cost trend for adversaries, as their proportions increase. However, as the proportion of adversaries increase, utility for both kinds of agents decreases as seen in Figure 9B and Figure 9D. Thus, while a small number of adversaries can get away by exploiting the cooperative nature of other agents, the decreasing utility trend creates a disincentive for adversaries to increase their proportion in the population.

**FIGURE 10:**
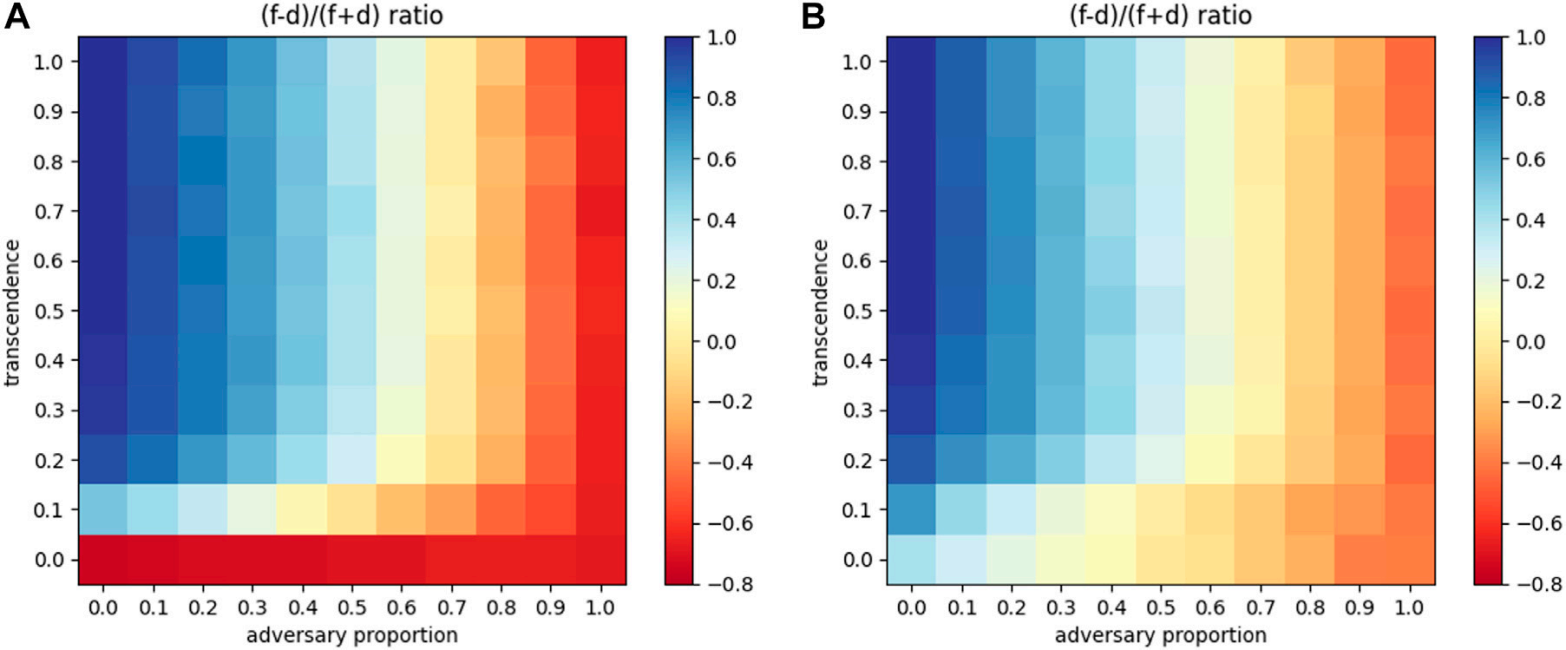
2D heatmap of adversarial proportion vs. transcendence for the ratio (*f*–*d*)/(*f* + *d*) **(A)** With attenuation **(B)** With attenuation and network elasticity.

We now vary both transcendence levels (along with distance updates), as well as the proportion of adversaries in the network, to see overall trends. These are presented as 2D heatmaps with the proportion of adversaries denoted on the *x*-axis and varying levels of transcendence on the *y*-axis as shown in Figure 10. Figure 10A depicts a network that only performs distance updates, while Figure 10B depicts a network that performs both distance and *γ* updates. Both networks are trained until *γ* values and distances stabilize. Then, the trained network is used to transmit 5000 packets to compute the proportion of packets forwarded and dropped. Each block in the 2D heatmaps represents the following expression: (*forwarded* − *dropped*)/*total* packets.

In the case shown in Figure 10A, on an aggregate, 58.68% more packets were forwarded than dropped, across the matrix, denoting a good level of resilience. With both distance and elasticity updates shown in Figure 10B shows that the forwarding of packets further improves to 67.77% more than dropping. Thus network elasticity accounts for a 10% increase in packet forwarding in the same network, highlighting the importance of curating elasticity of identity, in agents.

### 4.4 Baseline comparison

It is useful to compare how the model of computational transcendence fares in comparison to a baseline. As discussed, *reciprocity* is used as the baseline, where the players estimate the behaviour (packet forwarding probability) of their neighbours, and then reciprocate with similar behaviour.

Every player in the graph is initialized with a random packet forwarding probability. For a few rounds, players forward or drop the packets based on their initialized packet forwarding probability. Then they estimate the forwarding probability of their neighbours and adjust their forwarding probability accordingly, thus demonstrating reciprocity. Adjustment of forwarding probabilities stop when their updates fall below a threshold. After stabilization, a batch of test packets are sent, and the proportion of forwarded packets is calculated.

**FIGURE 11:**
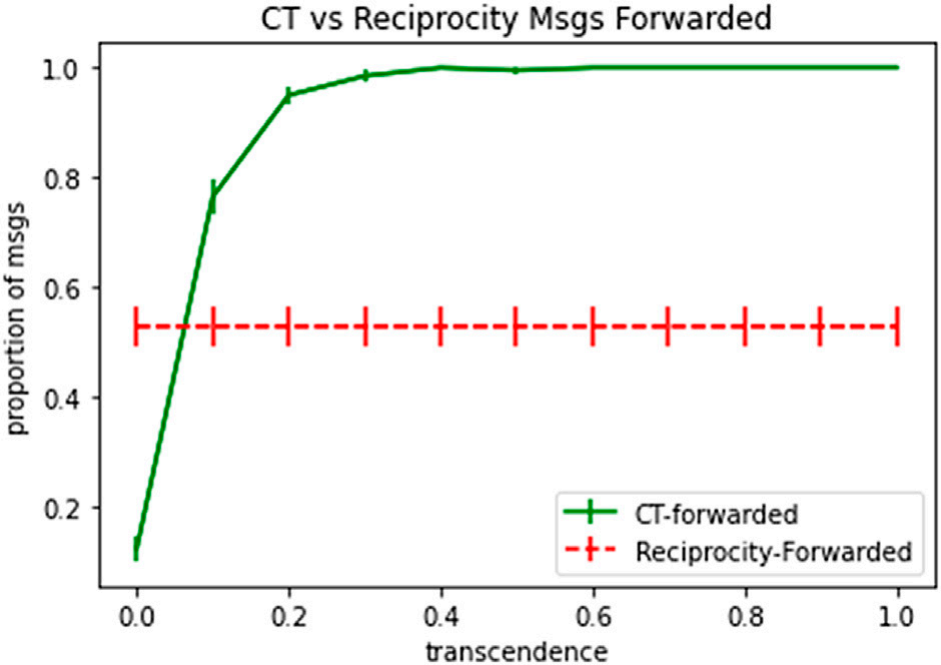
Proportion of packets forwarded in Computational Transcendence *vs*. Reciprocity.

Figure 11 shows the proportion of test packets forwarded in case of baseline model of reciprocity *vs.* proportion of test packets forwarded in case of computational transcendence, with varying level of transcendence denoted by the *x*-axis and with error bars denoting standard error. When forwarding probability of agents is initialized to uniformly distributed random values, reciprocity settles the network to a state where approximately 50% of the packets are dropped. Transcendence starts off with much lower levels of packet forwarding rates to begin with, but quickly reach 100% packet forwarding rates, as transcendence levels increase.

## 5 Discussion

The model of computational transcendence, as developed in this paper, represents a significant paradigmatic departure in modeling responsible autonomy. In this section, we reflect on some more pending issues. First, we compute a few analytic bounds for transcendence and then interpret a few other paradigms of responsibility, through the lens of transcendence.

Rational incentive for an agent to perform an action depends on the disparity between expected utility and cost of performing an action. In the model presented, we set the cost at a tenth of the payoff accrued, with the argument that an action is rational only if its payoff far exceeds the cost. Here, we parameterize this further, to ask how does elasticity relate to the difference between cost and payoff. For an intermediary, we first note the expected utility from forwarding or dropping its packet as given in Table 4, computed in Eq. 9.

We can see that a system of payoffs would make rational sense to participate in, when *E*(*f*) ≥ *E*(*d*). This leads us to the following inequality:

$$\gamma^{d\left(i,j\right)}\geq \frac{\kappa_{i}}{2*msgUtility}$$ (13)

Given that the highest value of *γ* is 1, this further leads us to a limiting condition of *κ*_*i*_ ≤ 2*msgUtility, for a transcendence-based solution to be feasible. Also, since the right hand side of Eq. 13 sets a lower bound on the value of *γ* ^*d*(*i*,*j*)^, this implies that when the intermediary cost *κ*_*i*_ is small compared to *utility* accrued by the source, even a small level of transcendence provides sufficient incentive for the agent to act responsibly.

We now compare the paradigm of transcendence, with other commonly occurring paradigms for promoting responsible behavior.

Cooperative behavior appears in different forms in ecology. A common paradigm of cooperation is *mutualism* Bronstein (2015), a term coined by Beneden Beneden (1876) in 1876, where species that are otherwise not dependent on one another, often cooperate to derive mutual benefit. Common examples of mutualism include the relationship of birds and bees with flowers and pollen. Disparate species discovering mutually beneficial activities may have been by accident, but it is not implausible to imagine some kind of identity transcendence between independent species that are strongly attracted towards one another.

Pro-social behavior–defined as the behavior to alleviate another’s need or improve their welfare Cronin (2012) may also be interpreted from the lens of transcendence. Apart from humans, many animals, primarily nonhuman primates, display pro-social behavior towards others of the same or other species in specific contexts. Humans and some animals are sensitive to others’ distress strongly enough to *act* in their welfare, leading to empathetic or pro-social behaviour Decety et al. (2016). Some examples of pro-social behavior include the following. Chimpanzees are known to help other chimpanzees by passing the right tools when the chimpanzee can visually see which tools are required by the other chimpanzee Yamamoto et al. (2012). Similarly, rats are known to display pro-social behavior by freeing another rat placed in a restrainer, if they had previously lived with a rat of same strain, and even if they were pitted against one another earlier Bartal et al. (2011, 2014).

We can interpret the above as familiarity–coming from even adversarial interactions, leading to some form of identity transcendence over time. While familiarity is ordinarily thought to breed contempt, familiarity is just as likely to breed empathy and transcendence.

## 6 Conclusions and future work

The elusive notion of our sense of self has long been considered a critical element in the innate sense of ethics and responsibility in humans. The model presented in this paper is our attempt to computationally model this elastic sense of self, to explore how it leads to responsible behavior. We demonstrate in game-theoretic scenarios like prisoners’ dilemma and collusion, computational transcendence can lead to responsible behaviour even in a one-shot game. Further, we experiment with different variations of the model using multi-agent framework which models dilemma between selecting responsible vs. irresponsible choices. Our simulation results show that autonomous agents with computational transcendence demonstrate adaptability and resilience. Finally, we also show that our model outperforms baseline model of reciprocity.

This paper presents the core model of computational transcendence developed for a static multi-agent network. Simulation results provide a baseline validation of the model. This model can be further extended and fine-tuned (for example by incorporating context specific entities and designing use-case specific utility functions) such that it can be applied to more realistic scenarios and in-turn be tested on use-case specific metrics. We conjecture that the proposed model involving distance and elasticity updates can seamlessly extend to dynamic networks like mobile ad hoc networks. We intend to test this in future endeavors. Also, in this version, agents’ identity sets comprise only of other agents in their immediate neighborhood, which can be extended to include agents further away, or other classes of objects. Finally, we plan to incorporate computational transcendence in application scenarios like supply chains, vehicular traffic management, etc.

We present computational transcendence primarily as a preliminary model with an intent to encourage more research interest in this direction, that has the potential to build versatile responsible autonomous AI systems.

## Data Availability

The raw data supporting the conclusion of this article will be made available by the authors, without undue reservation.
